# Metabolic health status and renal disorders: a cross-sectional study

**DOI:** 10.1038/s41598-023-48333-9

**Published:** 2023-11-27

**Authors:** Firouzeh Moeinzadeh, Mohammad Hossein Rouhani, Shiva Seirafian, Sahar Vahdat, Mojgan Mortazavi, Cain C. T. Clark, Farnaz Shahdadian

**Affiliations:** 1 Isfahan Kidney Diseases Research Center, Department of Internal Medicine, Isfahan University of Medical Sciences, Isfahan, Iran; 2 Department of Community Nutrition, School of Nutrition and Food Science, Isfahan University of Medical Sciences, and Food Security Research Center, Isfahan University of Medical Sciences, Isfahan, Iran; 3 Centre for Intelligent Healthcare, Coventry University, Coventry, CV1 5FB UK; 4 Department of Clinical Nutrition, School of Nutrition and Food Science, Isfahan University of Medical Sciences, Isfahan, Iran

**Keywords:** Medical research, Nephrology, Risk factors

## Abstract

Previous surveys suggests that body mass index (BMI) may be positively related to development of chronic kidney disease (CKD). However, this association might be altered by metabolic syndrome. Therefore, we aimed to evaluate the association of metabolic health status with CKD. The present cross-sectional study was carried out on 3322 representative sample of Iranian adults. Metabolic syndrome was identified based on the National Cholesterol Education Program (NCEP) Adult Treatment Panel III (ATP III) and BMI was assessed by anthropometric measurements. Estimated glomerular filtration rate (eGFR) was calculated by modification of diet in renal disease-Chronic Kidney Disease Epidemiology Collaboration (MDRD-EPI) formula. Subjects were categorized into four phenotypes: metabolically healthy normal weight (MHNW), metabolically healthy overweight and obesity (MHO), metabolically unhealthy normal weight (MUHNW), and metabolically unhealthy overweight and obesity (MUHO). Based on multivariate-adjusted models, the risk of CKD was significantly higher in MUHO compared with MHNW (OR: 1.48; p < 0.05). Although MUHNW and MUHO were associated with lower eGFR and albuminuria, the significant association was not observed in case of hematuria. Furthermore, subjects with kidney stones tended to be in MHO (OR: 1.42; p < 0.05) and MUHO phenotypes (OR: 1.64; p < 0.05), in comparison to the MHNW phenotype. The odds of kidney disorders were higher in adults with metabolic syndrome, regardless of BMI. However, this relationship might be strengthened by the concomitance of metabolic syndrome and obesity. To verify our findings, clarify the causality, and elucidate the underlying mechanisms, further research are warranted.

## Introduction

Chronic kidney disease (CKD) has been recognized as a gradual and progressive loss of renal function, present for at least 3 months, and is considered as a leading cause of end-stage renal disease (ESRD), as well as increasing the risk of morbidity and mortality from cardiovascular diseases^1,2^. CKD may be defined by abnormal urinary albumin excretion (at least 30 mg per 24 h or albumin to creatinine ratio at least 30 mg/g creatinine), decreased glomerular filtration rate (GFR) less than 60 mL/min/1.73 m^2^, and markers of kidney damage, including hematuria or structural abnormalities by imaging persisting for more than 3 months^1,3^.

The prevalence of CKD varies between 8–16% in populations from different parts of the world^4–6^. The major complications of impaired kidney functions are early CVD, anemia, metabolic acidosis, and bone diseases^5,7,8^; whilst the leading risk factors and predictors for CKD are impaired fasting plasma glucose, hypertension, and high body mass index (BMI)^6,9,10^.

Accumulating evidence has suggested that overweight and obesity may be implicated in development of CKD^11–14^. However, the association between BMI and the prevalence of CKD could be modified by metabolic health status, including presence or absence of insulin resistance, dyslipidemia, hypertension, as well as metabolic syndrome^13,15^. A study by Hanks et al. reported that the risk of mortality among adults with CKD was lower in the subgroup of overweight and obese individuals with normal metabolic status, called metabolically healthy overweight and obesity (MHO), compared with metabolically healthy normal weight (MHNW)^16^. In addition, Panwar et al. showed that the association between BMI and ESRD could be significantly modified by metabolic profiles; thereby indicating the higher BMI reduced the risk of ESRD in subjects without metabolic syndrome^17^. However, several studies have suggested that overweight and obese patients are not protected from CKD risk by healthy metabolic profiles^13,15^. Indeed, one study identified that the risk of CKD was higher in unhealthy metabolic patients, whether overweight and obese or normal-weight, compared with subjects with healthy metabolic status^18^. Furthermore, other studies did not found any significant association between MHO and CKD compared with normal-weight counterparts^19,20^.

Some systematic review and meta-analyses have reported that metabolically unhealthy subjects have an elevated risk for CKD, regardless of BMI and in subjects with healthy metabolic status, higher BMI is accompanied by an increased risk of kidney disorders^21–23^. However, this point should be taken into account that previous meta-analyses and their included studies that investigated the association between phenotypes of metabolic health status and renal disorders mostly focused on measuring eGFR to define CKD and renal disorders, and did not consider other markers of kidney damage, including hematuria, albuminuria or structural abnormalities.

Because of the contradictory results and dearth of studies that have evaluated the association between CKD and other types of kidney disorder in subtypes of BMI and metabolic profiles, we sought to conduct a cross-sectional studies to clarify the association of CKD and other kidney disorders with subtypes of metabolic health status and BMI.

## Materials and methods

### Study design and participants

The current cross-sectional population-based study was designed to be conducted in cooperation with Isfahan University of Medical Sciences. Participants were recruited from those visiting the health care centers within Isfahan city. The eligibility criteria included adults 18 years and above, living in Isfahan, willing to participate in study, lack of fever and common cold at the time of laboratory tests, refrained engaging in heavy exercise 48 h before laboratory tests, and not fasting. Those with incomplete questionnaires or not willing to participate in the tests were excluded. Also, pregnant women or those in their menstruation period were excluded. Finally, a total of 3322 eligible adults were included in the current study. This study was carried out in accordance with the Declaration of Helsinki and with the approval of the local Ethics Committee of Isfahan University of Medical Sciences (IR.MUI.REC.1396.1.086). All studied individuals provided written informed consent prior to study commencement**.**

### Data collection

#### Anthropometric measurement

Weight was measured by a mechanical scale (Zyklusmed ZYK-MS01, China), with 0.01 kg accuracy, with participants wearing minimal clothing and unshod. Also, a non-stretch tape (Seca) was applied for stature measurement, to the nearest 1 mm, and participants unshod. Body Mass Index (BMI) was computed as weight (kg)/height^2^ (m^2^).

#### Blood pressure and laboratory measurement

Blood pressure (BP) was evaluated within the medical examination, with participants in a sitting position, after a resting phase of at least 5 min. The subjects were asked to refrain from consuming tea and food, smoking, and exercise, for at least half an hour before BP measurement, and if the bladder is full, it should be emptied. Systolic and diastolic BPs (SBPs and DBPs) were measured using a digital sphygmomanometer (Omron BF511 (Omron Corp, Kyoto, Japan)) on the right arm. If the first BP was above 140/90 mmHg, a second measurement was carried out with a break of 15 min between measurements, and BP was defined as the mean of first and second measurements^24^.

Peripheral blood was obtained by means of venipuncture, after an overnight fast of at least 12 h from each subject. Albumin and creatinine were measured from a spot morning urine sample. Urinary Albumin was measured by using sulfosalicylic acid procedure (MN (analyticon) kit), whilst serum creatinine (mg/dl) and urine creatinine were determined by means of the Jaffè calorimetric method using a Hitachi-917 auto-analyzer (Pars Azmun kit). The urine ACR (UACR) was calculated by dividing urine albumin by urine creatinine and expressed as milligrams per gram. eGFR was calculated by Modification of Diet in Renal Disease (MDRD) study equation—Chronic Kidney Disease-Epidemiology Collaboration (CKD-EPI) equation^25^.

Other laboratory parameters were measured by enzymatic colorimetric method using Pars-Azmun kits on Hitachi-917 auto-analyzer, including fasting blood glucose (GOD-PAP), serum levels of total cholesterol, high-density lipoprotein cholesterol, low-density lipoprotein cholesterol (CHOD), and serum triglyceride (DOD-PAP). Blood Urea Nitrogen (BUN) was also measured using the Urease GLDH method on a Hitachi-917 analyzer (Adit kit).

### Definitions

A BMI equivalent to or higher than 25 was considered as overweight and obese and lower than 25 considered as non-obese, based on Asian-specific BMI criteria^26^.

The National Cholesterol Education Program (NCEP) Adult Treatment Panel III (ATP III) was applied for determination of metabolic syndrome. Subjects who met at least three of the following conditions were considered as metabolic syndrome: (1) elevated waist circumference (≥ 102 cm (≥ 40 inches) in men and ≥ 88 cm (≥ 35 inches) in women), (2) Elevated triglycerides (≥ 150 mg/dL (1.7 mmol/L) or on drug treatment for elevated triglycerides), (3) hypertension (≥ 130 mmHg systolic blood pressure or ≥ 85 mmHg diastolic blood pressure or on antihypertensive drug treatment in a patient with a history of hypertension), (4) hyperglycemia (≥ 100 mg/dL or on drug treatment for elevated glucose), and (5) Reduced HDL-C (< 40 mg/dL (1.03 mmol/L) in men and < 50 mg/dL (1.3 mmol/L) in women or On drug treatment for reduced HDL-C)^27,28^.

Based on BMI and metabolic health status, subjects were categorized into four groups: metabolically healthy overweight and obesity (MHO), metabolically healthy normal weight (MHNW), metabolically unhealthy overweight and obesity (MUHO), and metabolically unhealthy normal weight (MUHNW).

We defined CKD as decreased renal function for more than 3 months, determined by the eGFR based on the following criteria. The presence of less than 30 mg/g albumin, 30–299 mg/g creatinine, and equivalent to or greater than 300 mg/g creatinine in a random urine sample were classified as normal, moderately, and severely increased albuminuria, respectively^3^. Imaging techniques were performed for diagnosis of any unnamed disorder. Whole urine analysis was performed for urinary RBC count and hematuria diagnosis, and an RBC count > 2 per high power field was considered as positive^29^.

### Other assessments

By using an electronic questionnaire (http://www.ckd-epidemiology.ir), additional predictors of interest were assessed, including; gender, age, marital status, literacy, residence, smoking, opium and substance abuse, alcohol use, medications, self-reported history of cardiovascular diseases, diabetes, and hyperlipidemia, use of herbal medicines or preparations, and analgesics. In addition, habitual physical activity was evaluated by the Baecke questionnaire^30^.

### Statistical analysis

To evaluate the normality of quantitative variables, the Kolmogorov–Smirnov test was applied. The quantitative variables were illustrated as mean ± SD and qualitative variables as frequency (percentage). To compare quantitative variables across four categories of BMI and metabolic health status, one-way analysis of variance (ANOVA) was applied, while for categorical variables, Chi-square was used. The correlation of eGFR and UACR with anthropometric and biochemical variables across BMI and metabolic phenotypes was evaluated by Pearson correlation coefficients. To determine the association between phenotypes of BMI and metabolic status with CKD and related disorders, multivariable logistic regression was applied. The odds ratios (ORs) and their 95% confidence intervals (95% CIs) were calculated in crude and adjusted models. The MHNW phenotype was considered as the reference category in all analyses. SPSS software version 16 (IBM, Chicago, IL) was used to perform analysis and a p-value < 0.05 (two-sided) was, a priori, considered statistically significant.

## Results

Demographic and biochemical characteristics of the study participants across BMI and metabolic status phenotypes are demonstrated in Table 1. The percent of females in MUHNW was higher than other groups. Individuals with unhealthy metabolic status, whether normal weight or overweight and obese, were older than healthy metabolic subjects. Body weight, BMI, and waist circumference were lower in MHNW in comparison to other groups. The lipid profiles, blood pressure, and FBS tended to be abnormal in unhealthy metabolic subjects compared to metabolically healthy individuals, whether normal or abnormal in weight. In ACR, the highest levels were associated with the MUHO phenotype. Furthermore, eGFR in MHNW and MHO was greater in comparison to MUHNW and MUHO, respectively. The prevalence of albuminuria, CKD, and kidney stones were higher in metabolically unhealthy phenotypes compared to metabolically healthy counterparts. However, no significant differences in prevalence of hematuria were observed among phenotypes of BMI and metabolic status.

**Table 1:** Demographic and biochemical characteristics of study participants across combined BMI and metabolic syndrome.

Variables	Total	Metabolic health status				P-value^a^
		MHNW	MUHNW	MHO	MUHO	
Gender						< 0.001
Male	1355 (40.8)	464 (46.3)	36 (27.1)	578 (41.9)	277 (34.3)	
Female	1967 (59.2)	539 (53.7)	97 (72.9)	800 (58.1)	531 (65.7)	
Age (years)	49.39 ± 14.06	45.12 ± 15.18^2,3,4^	55.49 ± 13.30^1,3^	48.74 ± 13.16^1,2,4^	54.77 ± 12.01^1,3^	< 0.001
Education						< 0.001
Illiterate	439 (13.2)	93 (9.3)	28 (21.1)	151 (11)	167 (20.7)	
High school	1209 (36.4)	281 (28)	53 (39.8)	526 (38.2)	349 (43.2)	
Diploma	1036 (31.2)	355 (35.4)	35 (26.3)	440 (31.9)	206 (25.5)	
Bachelor	504 (15.2)	216 (21.5)	13 (9.8)	205 (14.9)	70 (8.7)	
Higher than bachelor	134 (4.0)	58 (5.8)	4 (3)	56 (4.1)	16 (2)	
Marital status						< 0.001
Single	295 (8.9)	159 (15.9)	13 (9.8)	93 (6.7)	30 (3.7)	
Married	2846 (85.7)	806 (80.4)	109 (82)	1221 (88.6)	710 (87.9)	
Death of wife	155 (4.7)	30 (3)	11 (8.3)	50 (3.6)	64 (7.9)	
Separated	26 (0.8)	8 (0.8)	0 (0)	14 (1)	4 (0.5)	
Body weight (kg)	72.48 ± 13.81	61.41 ± 8.98^3,4^	61.41 ± 7.65^3,4^	77.37 ± 11.62^1,2,4^	79.72 ± 13.39^1,2,3^	< 0.001
BMI (kg/m^2^)	26.88 ± 4.52	22.17 ± 2.09^2,3,4^	23.28 ± 1.42^1,3,4^	28.73 ± 3.23^1,2,4^	30.17 ± 3.74^1,2,3^	< 0.001
WC (cm)	93.32 ± 11.56	83.73 ± 8.68^2,3,4^	91.38 ± 7.35^1,3,4^	95.39 ± 9.82^1,2,4^	101.98 ± 9.25^1,2,3^	< 0.001
Waist to hip ratio	0.92 ± 1.19	0.86 ± 0.08^2^	1.43 ± 5.92^1,2,3^	0.89 ± 0.08^2^	0.94 ± 0.08^2^	< 0.001
SBP (mmHg)	120.7 ± 18.6	113.88 ± 17.24^2,3,4^	127.65 ± 17.68^1,3^	118.46 ± 16.77^1,2,4^	131.83 ± 18.02^1,3^	< 0.001
DBP (mmHg)	77.8 ± 11.28	74.61 ± 10.64^2,3,4^	80.37 ± 11.46^1,3,4^	76.62 ± 10.33^1,2,4^	83.31 ± 11.53^1,2,3^	< 0.001
TC (mg/dL)	171.46 ± 49.68	159.07 ± 48.88^2,3,4^	174.22 ± 53.76^1^	172.97 ± 46.32^1,4^	183.81 ± 51.88^1,3^	< 0.001
LDL-C (mg/dL)	96.49 ± 30.6	91 ± 31.1^3,4^	96.34 ± 31.25	98.48 ± 28.74^1^	99.94 ± 32.02^1^	< 0.001
HDL-C (mg/dL)	51.02 ± 11.09	53.61 ± 11.3^2,4^	45.64 ± 8.30^1,3^	52.58 ± 11.02^2,4^	46.03 ± 9.33^1,3^	< 0.001
TG (mg/dL)	154.32 ± 69.13	130.33 ± 62.86^2,3,4^	200.13 ± 99.03^1,3^	142.27 ± 57.78^1,2,4^	197.13 ± 65.70^1,3^	< 0.001
FBS (mg/dL)	90.23 ± 27.67	84.34 ± 20.15^2,4^	110.16 ± 45.72^1,3,4^	84.78 ± 16.93^2,4^	103.55 ± 38.46^1,2,3^	< 0.001
ACR (mg/g Cr)	18.85 ± 44.24	16.10 ± 34.90^4^	23.52 ± 42.91	16.74 ± 36.60^4^	25.08 ± 62.45^1,3^	< 0.001
eGFR (ml/min per 1.73 m^2^)	89.3 ± 20.5	93.63 ± 16.5^1,2,3^	84.23 ± 16.27^1,3^	89.6 ± 15.91^1,2,4^	84.39 ± 29.26^1,3^	< 0.001
Albuminuria						< 0.001
Yes	325 (10)	68 (6.9)	20 (15.6)	113 (8.4)	124 (15.7)	
No	2913 (90)	912 (93.1)	108 (84.4)	1228 (91.6)	665 (84.3)	
Hematuria						0.817
Yes	325 (9.8)	96 (9.6)	12 (9)	131 (9.5)	86 (10.6)	
No	2997 (90.2)	907 (90.4)	121 (91)	1247 (90.5)	722 (89.4)	
CKD						< 0.001
Yes	628 (18.9)	151 (15.1)	32 (24.1)	232 (16.8)	213 (26.4)	
No	2694 (81.1)	852 (84.9)	101 (75.9)	1146 (83.2)	595 (73.6)	
Metabolic syndrome						< 0.001
Yes	941 (28.3)	0	133 (100)	0	808 (100)	
No	2381 (71.7)	1003 (100)	0	1378 (1000	0	
Smoking						0.082
Yes	527 (15.9)	182 (18.1)	19 (14.3)	214 (15.5)	112 (13.9)	
No	2795 (84.1)	821 (81.9)	114 (85.7)	1164 (84.5)	696 (86.1)	
Alcohol consumption						0.055
Yes	118 (3.6)	48 (4.8)	2 (1.5)	45 (3.3)	23 (2.9)	
No	3202 (96.4)	954 (95.2)	131 (98.5)	1333 (96.7)	784 (97.1)	
Kidney stone						< 0.001
Yes	547 (16.5)	124 (12.4)	23 (17.3)	235 (17.1)	165 (20.4)	
No	2775 (83.5)	879 (87.6)	110 (82.7)	1143 (82.9)	643 (79.6)	
Sport index ()	2 ± 0.67	2.03 ± 0.7^4^	1.93 ± 0.63	2.04 ± 0.67^4^	1.91 ± 0.61^1,3^	< 0.001

^a^ Resulted from ANOVA for quantitative variables and Chi-square test for categorical variables.

Quantitative variables: mean ± SD.

Qualitative variables: frequency (percentage).

^1^Significant difference with MHNW.

^2^Significant difference with MUHNW.

^3^Significant difference with MHO.

^4^Significant difference with MUHO.

The correlation coefficients of eGFR and ACR with anthropometric and biochemical variables across BMI and metabolic phenotypes, and in the total study population, are shown in Table 2. In case of eGFR, a negative correlations were seen between eGFR and age, BMI, WC, ACR, blood pressure, FBS, and lipid profiles. In MUHNW group, the significant association was limited to age and FBS. The results for the ACR demonstrated positive significant correlations between ACR and age, BMI, WC, blood pressure, eGFR, and FBS, as well as a negative correlation with HDL-C.

**Table 2:** Pearson correlation analysis of eGFR and ACR with anthropometric and biochemical variables across metabolic health status.

Variables	Total		MHNW		MUHNW		MHO		MUHO	
	r	p	r	p	r	p	r	p	r	p
eGFR										
Age (years)	− 0.531	< 0.001	− 0.682	< 0.001	− 0.633	< 0.001	− 0.643	< 0.001	− 0.314	< 0.001
Body weight (kg)	0.002	0.917	0.002	0.946	0.058	0.518	0.133	< 0.001	0.103	0.004
BMI (kg/m^2^)	− 0.116	< 0.001	− 0.141	< 0.001	0.112	0.213	− 0.073	0.009	0.046	0.208
WC (cm)	− 0.147	< 0.001	− 0.214	< 0.001	0.043	0.635	− 0.069	0.015	0.009	0.802
Waist to hip ratio	− 0.013	0.465	− 0.206	< 0.001	0.003	0.975	− 0.076	0.007	− 0.021	0.557
SBP (mmHg)	− 0.244	< 0.001	− 0.256	< 0.001	− 0.206	0.022	− 0.236	< 0.001	− 0.144	< 0.001
DBP (mmHg)	− 0.132	< 0.001	− 0.123	< 0.001	− 0.019	0.836	− 0.173	< 0.001	− 0.009	0.805
ACR	− 0.129	< 0.001	− 0.133	< 0.001	0.066	0.474	− 0.161	< 0.001	− 0.115	0.002
TC (mg/dL)	− 0.079	< 0.001	− 0.176	< 0.001	0.010	0.909	− 0.056	0.045	0.031	0.388
LDL-C (mg/dL)	− 0.074	< 0.001	− 0.187	< 0.001	0.069	0.442	− 0.100	< 0.001	0.052	0.154
HDL-C (mg/dL)	0.038	0.034	0.052	0.113	0.030	0.741	− 0.042	0.133	− 0.031	0.396
TG (mg/dL)	− 0.061	0.001	− 0.072	0.030	− 0.024	0.788	0.026	0.361	0.042	0.249
FBS (mg/dL)	− 0.171	< 0.001	− 0.228	< 0.001	− 0.249	0.005	− 0.109	< 0.001	− 0.097	0.008
Sport index	0.006	0.740	− 0.025	0.450	0.067	0.463	− 0.016	0.576	0.009	0.802
ACR										
Age (years)	0.072	< 0.001	0.052	0.107	0.052	0.564	0.081	0.003	0.067	0.061
Body weight (kg)	0.024	0.176	0.032	0.314	0.131	0.141	− 0.017	0.523	0.023	0.524
BMI (kg/m^2^)	0.048	0.006	0.031	0.338	− 0.008	0.926	− 0.015	0.592	0.090	0.012
WC (cm)	0.068	< 0.001	0.073	0.023	0.072	0.419	0.019	0.492	0.104	0.004
Waist to hip ratio	0.002	0.894	0.075	0.020	− 0.033	0.708	0.055	0.045	0.043	0.232
SBP (mmHg)	0.101	< 0.001	0.083	0.009	0.175	0.049	0.094	0.001	0.107	0.003
DBP (mmHg)	0.065	< 0.001	0.036	0.263	0.183	0.040	0.082	0.003	0.037	0.303
eGFR	− 0.129	< 0.001	− 0.133	< 0.001	0.066	0.474	− 0.161	< 0.001	− 0.115	0.002
TC (mg/dL)	− 0.016	0.369	0.014	0.669	0.0	0.996	− 0.021	0.439	− 0.049	0.170
LDL-C (mg/dL)	− 0.022	0.206	− 0.008	0.806	− 0.032	0.720	0.015	0.585	− 0.090	0.012
HDL-C (mg/dL)	− 0.035	0.049	− 0.015	0.642	− 0.007	0.935	− 0.052	0.057	0.006	0.866
TG (mg/dL)	0.032	0.069	0.030	0.348	− 0.040	0.655	0.021	0.432	0.015	0.673
FBS (mg/dL)	0.083	< 0.001	0.070	0.028	0.125	0.159	− 0.001	0.970	0.134	< 0.001
Sport index	0.008	0.671	0.021	0.521	0.037	0.676	0.004	0.888	0.017	0.636

In Table 3, Multivariate adjusted odds ratio’s (OR) and 95% confidence interval’s (CI) for the association of BMI and metabolic health status with CKD and related disorders are shown. In CKD, the odds of having CKD were higher in MUHNW (OR: 1.78; 95% CI: 1.15, 2.75) and MUHO (OR: 2.02; 95% CI: 1.60, 2.55) in the crude model. After adjustment for potential confounders, the association remained significant only in the MUHO phenotype (OR: 1.48; 95% CI: 1.15, 1.89). No significant association was observed between hematuria and phenotypes of BMI and metabolic syndrome both in crude and adjusted models. In both crude and adjusted models, MUHNW and MUHO were associated with lower eGFR and albuminuria; however, no significant association was shown between MHO phenotype and eGFR and albuminuria. In addition, subjects with kidney stones tended to be in the MHO (OR: 1.45; 95% CI: 1.15, 1.84) and MUHO groups (OR: 1.81; 95% CI: 1.41, 2.34). Adjustment for confounding variables attenuated the association of kidney stones with MHO (OR: 1.42; 95% CI: 1.12, 1.81) and MUHO phenotypes (OR: 1.64; 95% CI: 1.26, 2.15).

**Table 3:** Multivariate adjusted odds ratio (OR) and 95% confidence interval (CI) for the association of metabolic health status with CKD and related disorders.

Metabolic health status	MHNW	MUHNW	MHO	MUHO
CKD (CKD vs. healthy)				
Crude model	1	1.78 (1.15, 2.75)	1.14 (0.91, 1.42)	2.02 (1.60, 2.55)
Model I^1^	1	1.28 (0.82, 1.99)	1.02 (0.81, 1.29)	1.52 (1.19, 1.94)
Model II^2^	1	1.29 (0.82, 2.02)	1.02 (0.81, 1.29)	1.51 (1.18, 1.93)
Model III^3^	1	1.30 (0.83, 2.04)	1.03 (0.82, 1.30)	1.48 (1.15, 1.89)
Hematuria (hematuria vs. healthy)				
Crude model	1	0.93 (0.49, 1.75)	0.99 (0.75, 1.30)	1.12 (0.82, 1.53)
Model I^1^	1	0.80 (0.42, 1.53)	0.95 (0.72, 1.27)	1.02 (0.74, 1.41)
Model II^2^	1	0.82 (0.43, 1.56)	0.96 (0.72, 1.27)	1.01 (0.73, 1.41)
Model III^3^	1	0.82 (0.43, 1.57)	0.98 (0.73, 1.30)	0.99 (0.71, 1.39)
eGFR (eGFR lower than 60 vs. higher than 60)				
Crude model	1	3.75 (1.78, 7.89)	1.35 (0.81, 2.26)	3.16 (1.93, 5.18)
Model I^1^	1	2.40 (1.07, 5.38)	1.48 (0.85, 2.59)	2.27 (1.32, 3.91)
Model II^2^	1	2.46 (1.09, 5.57)	1.48 (0.84 2.59)	2.27 (1.31, 3.91)
Model III^3^	1	2.52 (1.11, 5.72)	1.49 (0.85, 2.61)	2.34 (1.35, 4.07)
Albuminuria (ACR higher than 30 vs. lower than 30)				
Crude model	1	2.48 (1.45, 4.24)	1.23 (0.90, 1.68)	2.50 (1.83, 3.41)
Model I^1^	1	1.91 (1.10, 3.31)	1.13 (0.82, 1.56)	1.97 (1.42, 2.72)
Model II^2^	1	1.88 (1.08, 3.26)	1.12 (0.81, 1.54)	1.90 (1.37, 2.63)
Model III^3^	1	1.84 (1.06, 3.20)	1.09 (0.79, 1.50)	1.85 (1.33, 2.56)
Kidney stone (kidney stone vs. healthy)				
Crude model	1	1.48 (0.91, 2.41)	1.45 (1.15, 1.84)	1.81 (1.41, 2.34)
Model I^1^	1	1.40 (0.85, 2.31)	1.43 (1.13, 1.82)	1.68 (1.29, 2.20)
Model II^2^	1	1.38 (0.84, 2.28)	1.43 (1.12, 1.81)	1.67 (1.28, 2.17)
Model III^3^	1	1.38 (0.83, 2.82)	1.42 (1.12, 1.81)	1.64 (1.26, 2.15)

^1^Adjusted for age and sex.

^2^Additionally adjusted for age, sex, education, alcohol consumption, and smoking.

^3^Additionally adjusted for age, sex, education, alcohol consumption, smoking, and physical activity.

## Discussion

In the present cross-sectional study, a significant positive association was found between prevalence of CKD and MUHO phenotypes. In addition, decreased eGFR and albuminuria were associated with MUHNW and MUHO. The present study provides further evidence that MHO and MUHO individuals are at higher odds for developing kidney stones. However, no significant association was found between hematuria and phenotypes of combined BMI and metabolic syndrome.

Although the association of overweight and obesity with CKD has been reported in previous studies^31,32^, this association could be notably affected by metabolic syndrome^33^. Indeed, previous studies have suggested that the association of obesity with CKD might be attenuated by components of metabolic syndrome^34,35^. The Framingham Offspring Study, that was conducted on subjects without history of stage III CKD at baseline, suggested that the significant association between obesity and higher risk of KD could be rendered statistically insignificant after adjustment for CVD risk factors^14^. On the other hand, individuals with both high BMI and metabolic syndrome purportedly have a synergist effect on the development and progression of CKD, which is in line with our study^36^.

The inconsistent results in the literature for the association between MHO and MUHNW, as intermediate subgroups, have been reported by previous studies. In contrast to the current study, a prospective cohort study on young and middle-aged men and women reported that MHO phenotypes increased risk of CKD, and a lack of metabolic abnormities could not protect subjects from incidence of CKD^15^. In addition, two cohort studies demonstrated that both MHO and MUHNW were associated with elevated risk of CKD incidence^13,37^. Concordant with our findings, the results of several studies has illustrated that metabolic syndrome, regardless of BMI, is related to an elevated CKD risk^18,38^. In addition, the results of a cross-sectional study showed that MUHO, but not MHO, elevated the risk of CKD in middle-aged Chinese population^39^. To interpret these results the results of previous studies and the present analysis be considered; indeed, although the total body fat and BMI was the same in both MHO and MUHO, the values of waist circumference as visceral fat measurement as well as waist to hip ration was lower in MHO phenotypes compared with MUHO counterparts^40,41^. In addition, previous studies have showed that insulin resistance appeared to be lower in MHO in comparison to MUHNW^42^.

Numerous, multifaceted, biological mechanisms are involved in the association of obesity and metabolic syndrome with CKD and kidney damage. Previous studies have suggested that in individuals with obesity, abnormal lipid profiles^43^, alteration in inflammatory status, as well as hormonal factors, might be implicated in initiation and development of CKD. Empirical evidence from previous studies supports that inflammatory status, including high levels of IL-6, TNF-α, and CRP, may be associated with lower eGFR and initiation of kidney injury^44,45^. Hormonal factors, such as insulin resistance and increased levels of insulin^46^, higher levels of leptin^47^, as well as decreased levels of adiponectin, might play important roles in proteinuria and incidence of CKD^47,48^.

In nephrolithiasis, a cohort study reported that obesity, as an independent risk factor, was associated with renal stone incidence, regardless of metabolic health status^49^. The up-regulation of inflammatory cytokines, such as TNF-α and IL-6^50^, oxidative stress^51,52^, as well as alteration in chemistry of urine^53^, might be considered as the underlying mechanism for the association between obesity and nephrolithiasis.

Several limitations should be taken into account in interpreting the results of this study. We are unable to draw a causal relationship between indicators of CKD and associated factors due to the cross-sectional nature of the study. Another limitation of this study is the difficultly in generalizing the results, since it was conducted in Isfahan, Iran. Despite these limitations, the present study included a large representative sample of the adult population in Iran using multi-stage cluster random sampling approach and estimated the prevalence of CKD based on its stages. We have also provided novel, and supportive, evidence to advocate the identification of metabolic phenotypes.

## Conclusion

A significant positive association was found between prevalence of CKD and MUHO phenotype. In addition, decreased eGFR and albuminuria were associated with metabolic syndrome, regardless of BMI. The present study provides further evidence that MHO and MUHO individuals may be at higher odds for developing kidney stones. However, no significant association was found between hematuria and phenotypes of combined BMI and metabolic syndrome. To verify our findings, clarify the causality, and elucidate the underlying mechanisms, further research must be conducted.

## Data Availability

The datasets used and/or analyzed during the current study available from the corresponding author on reasonable request.
